# Supplementary material for: Sex Differences in Chronic Thromboembolic Pulmonary Hypertension. Treatment Options over Time in a National Referral Center
Source: J Clin Med. 2021 Sep 19;10(18):4251. doi: 10.3390/jcm10184251 (PMC8466098; doi:10.3390/jcm10184251)
Supplement: Supplementary file 1 [file jcm-10-04251-s001.zip › jcm-1376354-supplementary.pdf]

Table S1. CTEPH Patient Characteristics at Diagnosis by Sex.

CTEPH Patient Characteristics at Diagnosis by Sex (*n* = 453)				
	**N (%)**	**Women (*n* = 252)**	**Men (*n* = 201)**	**Statistical Significance (*p* Value)**
Age—years (median—IQr)	453 (100.0)	64.8 (49.7–73.7)	58.6 (45.8–68.5)	<0.001
Arterial hypertension (*n*/%)	453 (100.0)	107 (42.5)	77 (38.3)	0.371
Diabetes (*n*/%)	453 (100.0)	35 (13.9)	15 (7.5)	0.030
Dyslipidemia (*n*/%)	453 (100.0)	75 (29.8)	42 (20.9)	0.032
Current or past smoking habit(*n*/%)	453 (100.0)	54 (21.4)	93 (46.3)	<0.001
BMI (median—IQr)	407 (89.8)	28.4 (24.2-31.6)	27.8 (24.6-30.4)	0.340
Coronary artery disease (*n*/%)	453 (100.0)	10 (4.0)	15 (7.5)	0.106
Cancer history (*n*/%)	453 (100.0)	36 (14.3)	28 (13.9)	0.914
Functional class—NYHA (*n*/%)<br>I–II<br>III–IV	435 (96.0)	80 (32.8)<br>164 (67.2)	82 (42.9)<br>109 (57.1)	0.030
Hypercoagulability (*n*/%)	453 (100.0)	108 (42.9)	80 (39.8)	0.512
PE history	453 (100.0)	198 (78.6)	159 (79.1)	0.890
Six-minute walking test—distance (mean ± SD)	363 (80.1)	348.7 ± 7.7	415.4 ± 10.2	<0.001
NtproBNP–mg/dL (mean ± SD)	348 (76.8)	1409.6 ± 166.9	1241.7 ± 164.3	0.491
FEV1—% predicted (mean± SD)	314 (69.3)	87.6 ± 1.3	80.8 ± 1.5	<0.001
FVC—% predicted (median–IQr)	307 (67.8)	92.0 (75.0–99.0)	89.2 (76.5–98.7)	0.073

BMI = body mass index; DVT = deep vein thrombosis; FEV1 = forced expiratory volume in the first second; FVC = forced vital capacity; SD = standard deviation; PE = pulmonary embolism.

Table S2. CTEPH Patient Hemodynamic Parameters at Diagnosis by Type of Procedure and Sex (*n* = 453).

CTEPH Patient Hemodynamic Values at Diagnosis by Type of Procedure and Sex (*n* = 453)												
	PEA				BPA				MT			
	N (%)	Women	Men	*p* Value	N (%)	Women	Men	*p* Value	N (%)	Women	Men	*p* Value
mPAP—mmHg (median—Iqr)	235 (100.0)	46.0 (37.0–57.0)	44.0 (36.0–55.0)	0.223	91 (100.0)	50.0 (42.0–58.0)	49.0 (39.0–52.0)	0.122	127 (100.0)	40.0 (32.0–50.0)	45.0 (34.5–51.5)	0.516
RAP—mmHg (median—Iqr)	218 (92.8)	8.0 (5.0–12.0)	8.0 (5.5–12.0)	0.702	91 (100.0)	9.0 (6.0–11.0)	8.0 (6.0–11.0)	0.785	121 (95.3)	7.0 (4.0–11.0)	8.0 (6.0–11.0)	0.578
CI—L/min/m^2 (median—IQr)	216 (91.9)	2.3 (1.9–2.8)	2.4 (2.0–2.8)	0.785	87 (95.6)	2.3 (1.9–2.7)	2.1 (1.9–2.5)	0.381	112 (88.2)	2.4 (2.0–2.8)	2.5 (2.1–2.8)	0.735
PVR—WU (median—Iqr)	208 (88.5)	9.8 (6.4–13.9)	7.8 (5.5–10.5)	0.005	91 (100.0)	10.1 (7.5-15.0)	9.3 (6.0–12.5)	0.106	125 (98.4)	6.9 (4.7–11.0)	6.5 (5.0–9.6)	0.738

CI = cardiac index; mPAP = mean pulmonary artery pressure; PVR = pulmonary vascular resistances; RAP = right atrial pressure; WU = Wood Units.
